# Management of neonates at risk of early onset sepsis: a probability-based approach and recent literature appraisal: Update of the Swiss national guideline of the Swiss Society of Neonatology and the Pediatric Infectious Disease Group Switzerland

**DOI:** 10.1007/s00431-024-05811-0

**Published:** 2024-10-17

**Authors:** Martin Stocker, Flavia Rosa-Mangeret, Philipp K. A. Agyeman, Jane McDougall, Christoph Berger, Eric Giannoni

**Affiliations:** 1 Clinic of Pediatric Intensive Care and Neonatology, Children’s Hospital of Central Switzerland and University of Lucerne, Lucerne, Switzerland; 2 Neonatology and Paediatric Intensive Care Unit, Geneva University Hospitals and Geneva University, Geneva, Switzerland; 3 Department of Pediatrics, Inselspital, Bern University Hospital, University of Bern, Bern, Switzerland; 4 Department of Neonatology, Inselspital, Bern University Hospital, University of Bern, Bern, Switzerland; 5 Department of Pediatrics, Children’s University Hospital of Zurich and University of Zurich, Zurich, Switzerland; 6 Clinic of Neonatology, Department Mother-Woman-Child, Lausanne University Hospital and University of Lausanne, Lausanne, Switzerland

**Keywords:** Neonatal sepsis, Early onset sepsis, Antimicrobial stewardship, Antibiotic therapy, Guideline

## Abstract

In Switzerland and other high-income countries, one out of 3000 to 5000 term and late preterm neonates develops early onset sepsis (EOS) associated with a mortality of around 3%, while incidence and mortality of EOS in very preterm infants are substantially higher. Exposure to antibiotics for suspected EOS is disproportionally high compared to the incidence of EOS with consequences for future health and antimicrobial resistance (AMR). A safe reduction of unnecessary antibiotic treatment has to be a major goal of new management strategies and guidelines.

- Antibiotics should be administered immediately in situations with clinical signs of septic shock. *Group B streptococcus* (GBS) and *Escherichia coli* (*E. coli*) are the leading pathogens of EOS. Amoxicillin combined with an aminoglycoside remains the first choice for empirical treatment.
- Serial physical examinations are recommended for all neonates with risk factors for EOS. Neonates without any clinical signs suggestive of EOS should not be treated with antibiotics. In Switzerland, we do not recommend the use of the EOS calculator, a risk stratification tool, due to its unclear impact in a population with an observed antibiotic exposure below 3%.
- Not all neonates with respiratory distress should be empirically treated with antibiotics. Isolated tachypnea or respiratory distress starting immediately after delivery by elective caesarean section or a clearly assessed alternative explanation than EOS for clinical signs may point towards a low probability of sepsis. On the other hand, unexplained prematurity with risk factors has an inherent higher risk of EOS.
- Before the start of antibiotic therapy, blood cultures should be drawn with a minimum volume of 1 ml in a single aerobic blood culture bottle. This standard procedure allows antibiotics to be stopped after 24 to 36 h if no pathogen is detected in blood cultures. Current data do not support the use of PCR-based pathogen detection in blood as a standard method. Lumbar puncture is recommended in blood culture–proven EOS, critical illness, or in the presence of neurological symptoms such as seizures or altered consciousness.
- The accuracy of a single biomarker measurement to distinguish inflammation from infection is low in neonates. Therefore, biomarker guidance is not a standard part of decision-making regarding the start or stop of antibiotic therapy but may be used as part of an algorithm and after appropriate education of health care teams.
- Every newborn started on antibiotics should be assessed for organ dysfunction with prompt initiation of respiratory and hemodynamic support if needed. An elevated lactate may be a sign of poor perfusion and requires a comprehensive assessment of the clinical condition. Interventions to restore perfusion include fluid boli with crystalloids and catecholamines. Neonates in critical condition should be cared for in a specialized unit.
- In situations with a low probability of EOS, antibiotics should be stopped as early as possible within the first 24 h after the start of therapy. In cases with microbiologically proven EOS, reassessment and streamlining of antibiotic therapy in neonates is an important step to minimize AMR.

*Conclusion*: This guideline, developed through a critical review of the literature, facilitates a probability-based approach to the management of neonates at risk of early onset sepsis.

**Table:** 

**What is Known:** *• Neonatal exposure to antibiotics is disproportionally high compared with the incidence of early onset sepsis with implications for future health and antimicrobial resistance.*	
**What is New:** *• A probability-based approach may facilitate a more balanced management of neonatal sepsis and antibiotic stewardship.*

**What is Known:**

*• Neonatal exposure to antibiotics is disproportionally high compared with the incidence of early onset sepsis with implications for future health and antimicrobial resistance.*

**What is New:**

*• A probability-based approach may facilitate a more balanced management of neonatal sepsis and antibiotic stewardship.*

## Introduction

Most recommendations for neonates at risk for early onset sepsis (EOS) published more than 10 years ago were focused on early detection and prompt initiation of antibiotic therapy. These guidelines were mainly based on the assessment of clinical signs and risk factors. The approach was to start antibiotic therapy in every symptomatic neonate and in asymptomatic neonates with a high risk for EOS due to risk factors such as chorioamnionitis [1–6]. As clinical signs and risk factors for neonatal infection are non-specific, the vast majority of neonates started on antibiotic therapy do not have EOS. Therefore, a more balanced approach between effective sepsis management and antimicrobial stewardship (AMS) is needed. Nevertheless, an early detection of bacterial infection remains the cornerstone.

The purpose of this publication is to thoroughly evaluate and reassess the existing body of knowledge and to update the Swiss national guidelines published in 2013 [7]. We are using the word EOS to describe the continuum of bacterial and non-bacterial infection, infection with organ dysfunction (defined as sepsis in other age groups), and septic shock. Over the past 10 years, several countries have revised their guidelines, and these will be used as a basis and benchmark [1–6, 8]. The goal is to develop a management framework that can be implemented broadly. This document covers the assessment, early detection, and management of EOS. This includes diagnostic procedures, deciding when to start empirical antibiotic treatment, early interventions to prevent and treat organ dysfunction, and determining the appropriate duration of antibiotic therapy. The emphasis is not limited to term and late preterm infants but extends to newborns of all gestational ages. Over 90% of infants developing microbiologically proven EOS are symptomatic during the first 48 h. Therefore, we focus our management strategies on neonates within the first 2 days of life.

## Probability-based recommendations for the management of neonates at risk of *EOS*

Management guidelines for EOS have shown a significant evolution over time. In the past, there was a notable variability among different international guidelines, with many lacking a consensus on relevant topics [9]. However, recent years have seen a shift towards greater alignment, yet inconsistencies and ambiguities remain in some recommendations, such as the use of the EOS calculator or serial physical examinations [3, 6]. This evolution reflects the ongoing efforts to refine and improve the management strategies for EOS, adapting to new research findings and clinical practices.

Moreover, most guidelines have targeted specific groups, often categorized by gestational age, primarily addressing term and/or late preterm neonates [3–5]. The American Academy of Pediatrics (AAP) has further added to this landscape by issuing three different sets of guidelines: one for preterm infants below 35 gestational weeks, one for those above 35 weeks, and another for neonates born to GBS-positive mothers [1–3]. Additionally, the National Institute for Health and Care Excellence (NICE) has published an extensive guideline, spanning 60 pages, which, while comprehensive, adds to the complexity and presents challenges in terms of quick comprehension and application in clinical settings [6]. These examples highlight the ongoing struggle to balance detailed, specific guidance with the need for clarity and practicality in EOS management.

It is important to note that no management strategy can identify at birth all newborns who will develop EOS. It is not possible to avoid some amount of overtreatment due to the margin of safety needed not to miss a true sepsis case. With a probability-based approach, it may be possible to balance effective sepsis detection and care and AMR [10]. Therefore, the zero-risk approach treating all neonates with at least some risk to develop EOS needs to be adapted by a balanced AMS culture. The aim is to early and effectively treat neonates with an ongoing bacterial infection early to achieve optimal short- and long-term outcomes but to expose fewer neonates to antibiotics.

In the process of developing new guidelines, it is essential to take into account local conditions, emphasize the importance of educational initiatives, and address the challenges associated with change management. Adopting a gradual approach, where changes are implemented in small, manageable steps, is often more practical and effective. Considering this, we kept important and established elements of the 2013 Swiss national guidelines while adapting it to decrease antibiotic use in low-risk situations [7].

## Critical appraisal of new evidence within the last decade

### Epidemiology

Several high-income countries have reported a decline in the incidence of EOS over the last 20 years [11–15]. The incidence of EOS is highest in extremely preterm infants (up to 13.5 per 1000 very preterm infants) and decreases with advancing gestational age [16–18]. In a retrospective study including more than 750,000 late preterm and term neonates in Europe, Australia, and North America, the incidence of EOS was 0.49 cases per 1000 live births with a range of 0.18 and 1.45 across networks [19]. The incidence of EOS in children born in Switzerland between 2012 and 2015 was 0.28 per 1000 live births in a prospective cohort study [18]. The severity of culture-proven EOS was considerable with septic shock in 26%, and a mortality of 18%, which is comparable with other studies [12, 20]. Yet, 43% of cases of EOS occurred in infants born at term and were associated with a low mortality rate of 3.2% [18]. Mortality rates in preterm infants with EOS are reported to be around ten times higher [12, 21, 22].

Conclusion: In Switzerland and other high-income countries, one out of 3000 to 5000 term and late preterm neonates develops EOS with a relatively low associated mortality of around 3%. Incidence and mortality of EOS in very preterm infants are substantially higher.

### Antimicrobial exposure and its effects in neonates

Exposure to antibiotics within the first week of life is disproportionally high compared to the incidence of EOS [19]. In a large network-based study in Europe, Australia, and North America, between 1.2 and 12.5% of late preterm and term neonates were started on antibiotic therapy leading to an exposure of 135 (range 54–491) antibiotic days per 1000 live births [19]. In Switzerland, the rate of antibiotic exposure for late preterm and term neonates was between 2.5 and 3%. This overexposure to antibiotics at the beginning of life represents a high burden for the health of future generations [10, 23]. Antibiotic therapy perturbates the development of the individual microbiome with possible long-term consequences [10, 23–28]. An antibiotic course of 48 h within the first days of life leads to a significant change in the microbiome, still measurable at 1 year of age [29]. Moreover, several studies have described an association of antibiotic therapy in early life with chronic diseases as obesity, asthma, diabetes, juvenile arthritis, celiac, and inflammatory bowel disease later in life [29–31]. Whereas these studies are all retrospective with an inherent bias, some human and animal models give an insight regarding possible mechanisms involved in the interaction between microbiome and future diseases [24, 32, 33]. In preterm infants, studies showed an association of prolonged duration of antibiotic therapy with increased rates of necrotizing enterocolitis, bronchopulmonary dysplasia, late-onset sepsis, and death [34–36]. In addition, antibiotic therapy may lead to increased antimicrobial resistance (AMR) with the consequence that broad-spectrum antibiotics may be increasingly needed in neonatal care [29, 37–39]. Increased AMR is a major global health threat with more than 1.2 million deaths worldwide directly attributed to drug-resistant infections [40].

Conclusion: Exposure to antibiotics for suspected EOS is disproportionally high compared to the incidence of EOS with consequences for future health and AMR. A safe reduction of unnecessary antibiotic treatment is an important goal of new management strategies and guidelines.

### Risk factors

Following the implementation of universal screening policies for GBS and intrapartum antibiotic prophylaxis (IAP), maternal fever as the hallmark of chorioamnionitis has become the major risk factor for EOS [41]. However, the term chorioamnionitis has been used to label a heterogeneous group of conditions characterized by infection and/or inflammation with variable consequences for the newborn [42]. A consensus conference from the American College of Obstetricians and Gynecologists, the AAP, and the Society for Maternal–Fetal Medicine provided updated definitions that may distinguish isolated maternal fever from suspected and proven intrauterine infection or inflammation [42]. Instead of chorioamnionitis, the term “triple I” (intrauterine inflammation, infection, or both) was introduced to improve the categorization of such cases into (i) isolated maternal fever (not triple I), (ii) suspected, and (iii) confirmed triple I [43].

IAP for maternal GBS colonization is highly effective. In a cohort study of more than 7600 deliveries, IAP given at least 4 h before delivery was effective in 91% of cases to prevent GBS EOS, and when given less than 4 h before delivery still had some protective effect [44]. It is important to note that not all risk factors have the same “weight,” and a combination of risk factors may have more than an additive effect. Gestational age is the strongest single predictor of EOS. Therefore, preterm infants below 34 weeks of gestational age need to be assessed thoroughly including clinical information from obstetricians and midwives to explore important coexisting risk factors such as suspected or proven triple I.

Conclusion: Isolated maternal fever is not synonymous with chorioamnionitis. In contrast to the previous guidelines published in 2013, infants born to GBS-positive mothers exposed to adequate intrapartum antibiotic prophylaxis (IAP) are not considered to be at high risk of developing EOS.

### Pathogens of *EOS* and empirical treatment

GBS and *Escherichia coli* are the leading pathogens of EOS, where the proportion of EOS cases caused by *E. coli* compared to GBS is increasing, especially in preterm infants [12]. Additionally, viridans group streptococci, enterococci, *Staphylococcus aureus*, *Klebsiella* species and other Enterobacterales, *Listeria monocytogenes*, and *Candida* spp. may cause EOS [12]. Empirical treatment should include amoxicillin in combination with an aminoglycoside, gentamicin, or amikacin. Due to good penetration of the blood–brain barrier, cephalosporins of the 3rd and 4th generation (cefotaxime, ceftazidime, or cefepime) should be considered in addition for suspected or proven meningitis with Gram-negative pathogens or septic shock [45]. In newborns with clinical signs suggestive of progression to septic shock, antibiotics should be started immediately after the collection of blood cultures [46]. In situations with ambiguous clinical signs, neonates need to be evaluated and observed, but antibiotics do not always need to be started immediately [46]. Besides bacterial EOS, non-bacterial EOS caused by fungal or viral infections has to be considered in specific situations. Congenital candidosis, a rare condition with known but non-specific risk factors such as prematurity, cerclage, rupture of membranes, and maternal vaginal candidosis, can present with skin lesions and progress to sepsis and septic shock requiring rapid initiation of systemic antifungals [47, 48]. EOS caused by Herpes simplex virus is rare but has to be considered in specific situations, and treatment with acyclovir has to be started early.

Conclusion: In situations with clinical signs of a septic shock, antibiotics should be administered immediately. GBS and *E. coli* are the leading pathogens of EOS. Amoxicillin with an aminoglycoside is the first choice for empirical treatment.

### *EOS* calculator

The EOS calculator is a risk stratification tool with an integrated guideline regarding the start of antibiotics aiming to reduce unnecessary antibiotic use. Several studies were published within the last decade describing the implementation and use of the EOS calculator. The EOS calculator predicts the risk of developing EOS based on four risk factors (gestational age, highest maternal antepartum temperature, duration of rupture of membranes, maternal GBS colonization) and one protective factor (type and timing of maternal intrapartum antibiotics) and can be adjusted to the local incidence of EOS [49]. In addition, the EOS calculator also serves as a guideline and proposes a clinical recommendation to observe or to treat with antibiotics according to the estimated risk of sepsis adjusted to observed clinical signs. The threshold to recommend the start of antibiotic therapy was set at the probability to develop EOS in 3 out of 1000. The use of the sepsis calculator successfully reduced exposure to antibiotics from a range of 5 to 15% of all late preterm and term newborns to a range of 3 to 5% [49, 50]. The impact of this approach in settings with lower antibiotic treatment rates, such as shown in different networks in Europe including Switzerland, is unclear and may increase antibiotic exposure [19]. Importantly, the purpose of the sepsis calculator is not to identify all cases of EOS and information on the safety of this approach in different settings is still limited [11, 51, 52].

Conclusion: In Switzerland, we do not recommend the use of the EOS calculator due to the unclear impact of this tool in a population with an observed antibiotic exposure below 3%.

### Serial physical examinations

More than 90% of neonates developing EOS become symptomatic within the first 48 h of life, irrespective of IAP [53, 54]. The strategy to withhold antibiotics based on serial physical examinations in well-appearing neonates at risk for EOS has been recommended in past guidelines in Italy and Switzerland [7, 55]. The presence of risk factors helps to decide which neonates need to be closely observed (Table 1). Serial observations entail evaluating respiration, heart rate, temperature, peripheral perfusion, and skin color at least every 4 h. Implementation of serial physical examination to guide empiric treatment has reduced antibiotic exposure, without delaying antibiotic treatment of infected neonates [56–60]. The possibility to perform serial physical examinations on the postnatal maternity wards without separating the infant from its mother is a big advantage of this approach but presents a challenge in case of standardized early discharge to home.

**Table 1:** Clinical signs and risk factors potentially related to neonatal early onset sepsis (EOS); definition of low probability of sepsis, serial, and continuous observation; start of antibiotics (compare Fig. 1)

Risk factors	• Maternal group B streptococcus (GBS) colonization (vaginal/rectal swab: current or previous, bacteriuria) with intrapartum prophylaxis missing or inadequate and without elective caesarean section before the onset of labor and intraoperative rupture of membranes • Preterm birth • Prolonged rupture of membranes > 18 h • Suspected triple I (maternal fever > 38 °C plus any of the following symptoms: maternal WBC > 15 G/l in the absence of corticosteroids, baseline fetal tachycardia, purulent fluid from the cervical os) • Confirmed triple I (suspected triple I plus any of the following: amniocentesis-proven infection, low glucose in amniotic fluid, placental pathology)
Clinical signs	• Tachypnea, respiratory distress, apnea • Tachycardia/bradycardia, poor peripheral perfusion, mottling • Temperature instability • Lethargy, irritability, muscular hypotonia, seizures • Vomiting, poor feeding
Low probability of sepsis	The following situations point towards a low probability of sepsis (assessment by specialist neonatologist/pediatrician): • Elective caesarean section (no rupture of membranes or contractions • Isolated tachypnea • Respiratory distress starting immediately after delivery without risk factor • Other explanation for observed clinical signs • Rapid resolution of clinical signs within a few hours
Serial observation	At the mother–child unit, monitor vital signs every 4 h for a total of 48 h: respiration, heart rate, temperature, peripheral perfusion, skin color
Continuous observation	At a specialized neonatology unit, monitoring continuously vital signs and clinical signs potentially related to infection, biomarker measurements according to local policy
Start of antibiotics	Blood culture of at least 1 ml needs to be taken before the start of antibiotics. Empirical treatment should include amoxicillin in combination with an aminoglycoside (gentamicin or amikacin). Prematurity due to chorioamnionitis or unexplained prematurity with the requirement for invasive ventilation and hemodynamic support with catecholamine are the main risk factors for death during EOS. In such scenarios, prompt initiation of antibiotics (within < 1 h) and support of vital functions are essential

Conclusion: Neonates without any clinical signs suggestive of EOS should not be treated with antibiotics. In Switzerland, we recommend to keep the strategy of serial observations for asymptomatic infants with risk factors for EOS.

### Scenarios with a low probability of *EOS*

In the previous guideline, the recommendation was to treat all infants with clinical signs, regardless of an additional assessment of the probability of EOS. The justification for this approach was that clinical signs potentially associated with EOS are non-specific. In a retrospective cohort study after the implementation of an AMS initiative withholding antibiotic treatment in neonates with isolated respiratory distress without other risk factors in the USA, the rate of empirical treatment of newborns with respiratory distress was reduced from 95 to 41% without missing a true EOS case [61]. It is important to note that the design and power of this study is insufficient to prove the safety of this approach. Nevertheless, the updated European guidelines on the management of respiratory distress syndrome highlight the importance of not treating all neonates with respiratory distress with antibiotics [62]. Cardiovascular signs such as impaired perfusion (mottled skin, prolonged capillary refill time, increased central-peripheral temperature difference), tachycardia, and hypotension have the highest diagnostic value among clinical signs for EOS [63, 64].

Whereas it is difficult to define a list of scenarios with a high risk of EOS, it may be possible to identify some situations with a low probability of EOS. Neonates delivered via elective caesarean section without prior rupture of membranes or contractions exhibit a low risk of sepsis. This is also true for neonates who present with isolated tachypnea or respiratory distress immediately following delivery, especially when an alternative cause for these symptoms is clearly identified (i.e., pneumothorax). Whereas respiratory distress in preterm infants is expected due to prematurity, situations with unexplained prematurity and additional risk factors such as triple I have an inherent higher risk of EOS. Unexplained prematurity or explained by triple I with a requirement of invasive ventilation and hemodynamic support with catecholamines are the main risk factors for death during EOS [18].

Conclusion: Not all neonates with respiratory distress should be empirically treated with antibiotics. Neonates delivered via elective caesarean section, with an isolated tachypnea or respiratory distress starting immediately after delivery, or a clearly assessed alternative explanation than EOS for clinical signs may have a low probability of sepsis. On the other hand, unexplained prematurity and additional risk factors have an inherent higher risk of EOS.

### Blood and cerebrospinal fluid cultures

Detection of pathogens in blood cultures has improved due to automated blood culture systems and a better performance to detect low levels of bacteremia with a shorter time to positivity [65]. Blood cultures with a minimum of 1 ml blood sampled, primarily in an aerobic so-called pediatric bottle, before the start of antibiotic therapy have an excellent sensitivity [66–69]. Multifaceted interventions including education, guidelines, and feedback can increase compliance, optimize blood collection for culture, and improve sensitivity [70]. In a study of 594 bacteremic EOS episodes conducted in the USA, pathogens grew within 36 h of incubation in over 94% of episodes, regardless of maternal antibiotic administration [71]. These results were confirmed in other populations [72–74]. Therefore, the traditional 48-h period of empiric antibiotic treatment can be safely decreased to 24–36 h [75, 76].

To replace or increase the sensitivity and specificity of pathogen detection in blood, PCR-based methods to increase the yield of pathogen detection have been studied with mixed results [77–80]. Currently, PCR as a standard evaluation for suspected EOS cannot be recommended due to limited data on accuracy. On the other hand, secondary use of PCR in cases with a high suspicion of culture-negative EOS due to critical illness and negative blood cultures may help to evaluate and manage the situation [79, 81].

Lumbar puncture is recommended in blood culture–proven EOS, critical illness, or in the presence of neurological symptoms such as seizures or altered consciousness. It should be considered and thoroughly discussed in all cases of suspected EOS [82]. The accuracy of biomarker-guided decision-making regarding the need to perform a lumbar puncture is low [83, 84]. If the neonate’s condition does not permit the safe performance of a lumbar puncture prior to starting antibiotics, the procedure can be deferred. In such cases, cell count and PCR to search for bacteria in the cerebrospinal fluid (CSF) can be performed when the neonate is stable to inform and guide further management [82].

Conclusion: Before the start of antibiotic therapy, blood cultures must be drawn with a minimum volume of 1 ml in an aerobic blood culture bottle. This standard procedure allows antibiotics to be stopped after 24 to 36 h if no pathogen is detected in blood cultures and clinical circumstances allow. Lumbar puncture is recommended in blood culture–proven EOS, critical illness, or in the presence of neurological symptoms such as seizures or altered consciousness. Current data do not support the use of PCR-based methods in blood as a standard method to improve the accuracy of pathogen detection in suspected EOS cases.

### Biomarkers

Biomarkers have been used by clinicians for a long time with the aim to improve decision-making regarding the management of EOS. Only a small number of prospective studies with a sufficiently large sample size and biomarker implemented in an algorithm were published [85]. The most commonly used biomarkers are white blood count (WBC), C-reactive protein (CRP), interleukin 6 (IL-6), and procalcitonin (PCT) [86–90]. There is only one large randomized controlled trial using biomarker-guided decision-making to shorten the duration of empirically started antibiotic therapy in newborns [86]. Whereas analyses using machine learning underline the possible benefit of biomarkers, some published studies reported a risk for prolonged antibiotic therapy due to the implementation of biomarkers into decision-making for the management of EOS [56, 63, 91–93]. Recently, novel approaches like RNA signatures have been used in research settings, with the added benefit of combining several biomarkers for the categorization of patients [94, 95].

Overall, no biomarker has shown acceptable accuracy regarding the diagnosis of culture-proven EOS, and routine measurements of biomarkers as a standard procedure to diagnose EOS are not recommended [85]. Nevertheless, unexplained leukopenia (below 5 G/l) or elevated CRP, PCT, or IL-6, as well as signs of organ dysfunction such as elevated lactate, low platelets, or prolonged INR together with suspected infection, are associated with bacterial sepsis [96].

Serial negative PCT or CRP measurements may serve as an additional supporting argument for discontinuing antibiotic therapy in late preterm and term infants within 24 to 36 h. Normal values for PCT depend on age, and the most used target values for CRP are below 10 to 15 mg/l [85–87, 90]. The implementation of a biomarker-guided algorithm needs education and a change management of staff to prevent potential adverse consequences such as prolonged antibiotic therapy due to increased PCT or CRP values.

Conclusion: The accuracy of a single biomarker measurement to distinguish inflammation from infection is low. Therefore, biomarker guidance is not a standard part of decision-making regarding the start or stop of antibiotic therapy but may be used as part of an algorithm and after appropriate education of the local health care team.

### Organ dysfunction in *sepsis*

Since 2016, sepsis is defined, in adults, as a life-threatening organ dysfunction caused by a dysregulated host response to infection [97]. A consensus statement published in 2024 defined pediatric sepsis and septic shock accordingly [98]. Neonates are excluded in this new consensus statement, and an updated, international consensus definition for neonatal sepsis is still lacking [99, 100]. Newborns that die from bloodstream infection have a failure of respiratory and cardiovascular systems, which underlines the importance of including parameters of organ dysfunction in clinical assessment [18, 101]. A blood gas analysis with lactate is recommended in all neonates started on antibiotics to evaluate cardiovascular dysfunction in addition to clinical monitoring. If clinical signs of poor perfusion or an elevated lactate are observed, a fluid bolus of 10 ml/kg crystalloids should be administered immediately. If shock symptoms persist or lactate is not clearly decreasing, the start of a vasoactive agent, echocardiography, and care in a tertiary center are recommended [46]. In all cases, closely monitor respiratory rate, oxygen saturation, heart rate, temperature, blood pressure, and urine output. It is important to note that low blood pressure can be a late sign in septic shock in neonates. Chest X-ray may give important additional information for neonates in need of respiratory support. Blood coagulation studies are recommended in cases with septic shock, active bleeding, or low platelets. Investigations have to be repeated after specific therapy has been administered or if the organ dysfunction is worsening [46]. Organ dysfunction may be described and followed up using the neonatal Sequential Organ Failure Assessment (nSOFA) score which provides a reliable assessment for mortality in neonates with EOS [102].

Conclusion: Every newborn started on antibiotics should be assessed for organ dysfunction (respiratory, cardiovascular, and hematological dysfunction) with prompt initiation of respiratory and hemodynamic support if needed. An elevated lactate may be a sign of poor perfusion and requires a comprehensive assessment of the clinical condition. Interventions to restore perfusion include fluid boli with crystalloids and catecholamines. Care in a tertiary center is mandatory in case of critical illness.

### Reassessment of antibiotic therapy

If EOS is unlikely based on the course of clinical signs or there is an alternative explanation, clinicians should consider to stop antibiotic therapy at any time point to reduce unnecessary antibiotic exposure [10]. To be able to stop antibiotic therapy early in situations with a low probability of EOS, serial assessments of the neonate are mandatory.

An important role regarding the assessment of continuation of antibiotic therapy lies in the dynamic course of clinical signs. It is unlikely that antibiotic therapy leads to the resolution of clinical signs due to invasive bacterial infection within a few hours and this fast resolution might point towards no infection, especially if an alternative explanation for the clinical presentation exists. Potential signs of infection within hours after delivery may be caused by a variety of diseases and conditions other than infection. On the other hand, the progression of clinical signs within the first 24 h after the start of antibiotics may point towards infection. Nevertheless, it is imperative to closely monitor and re-evaluate the patient for alternative explanations than sepsis.

After the detection of the causative pathogen, streamlining antibiotics is an important step to prevent the emergence of antimicrobial resistance (AMR). For questions regarding streamlining and duration of antibiotic therapy, consultation with infectious disease specialists should be considered in cases of culture-positive EOS.

Recent publications investigated the switch from parenteral to oral antibiotics for neonates in good general condition with negative cultures [103, 104]. In the RAIN study, an early switch from parenteral to oral amoxicillin with clavulanic acid in late preterm and term neonates with suspected EOS was not associated with an increased incidence of adverse outcomes [103]. Investigators in Denmark published similar results in a population-based, multicenter study with an overall low rate of 1.5% of term neonates started on antibiotics [104]. In the investigated population, exposure to antibiotics was not increased with the possibility to switch to oral therapy, but the duration of hospitalization was shortened. Nevertheless, the key question persists: Were these neonates truly infected, and would discontinuing antibiotic therapy have yielded the same outcomes as administering oral antibiotics?

Conclusion: In situations with a low probability of EOS, antibiotics should be stopped anytime as early as possible. In cases with microbiologically proven EOS, streamlining antibiotic therapy is an important step to optimize treatment and minimize AMR. In cases with a high suspicion for culture-negative sepsis, antibiotics should be discontinued latest after 5 days and not continued orally.

## Comprehensive guidelines for the management of *EOS*

Implementation of new guidelines has to be accompanied by an educational intervention including knowledge transfer as well as elements of change management. Whereas clarity and recommendations are high in some parts of the guideline, there remains room for local adaptation in other parts (for example, the use of biomarkers). Professionals in charge of neonatal care have to ensure implementation and compliance at their own institution. To evaluate the effect of the recommended guidelines, we have to measure future performance with at least a minimal dataset [10]. The minimal dataset used in the AENEAS study, describing the population, rate of culture-positive EOS, and exposure to antibiotics within the first week of life, may serve as an example [19].

**Fig. 1:**
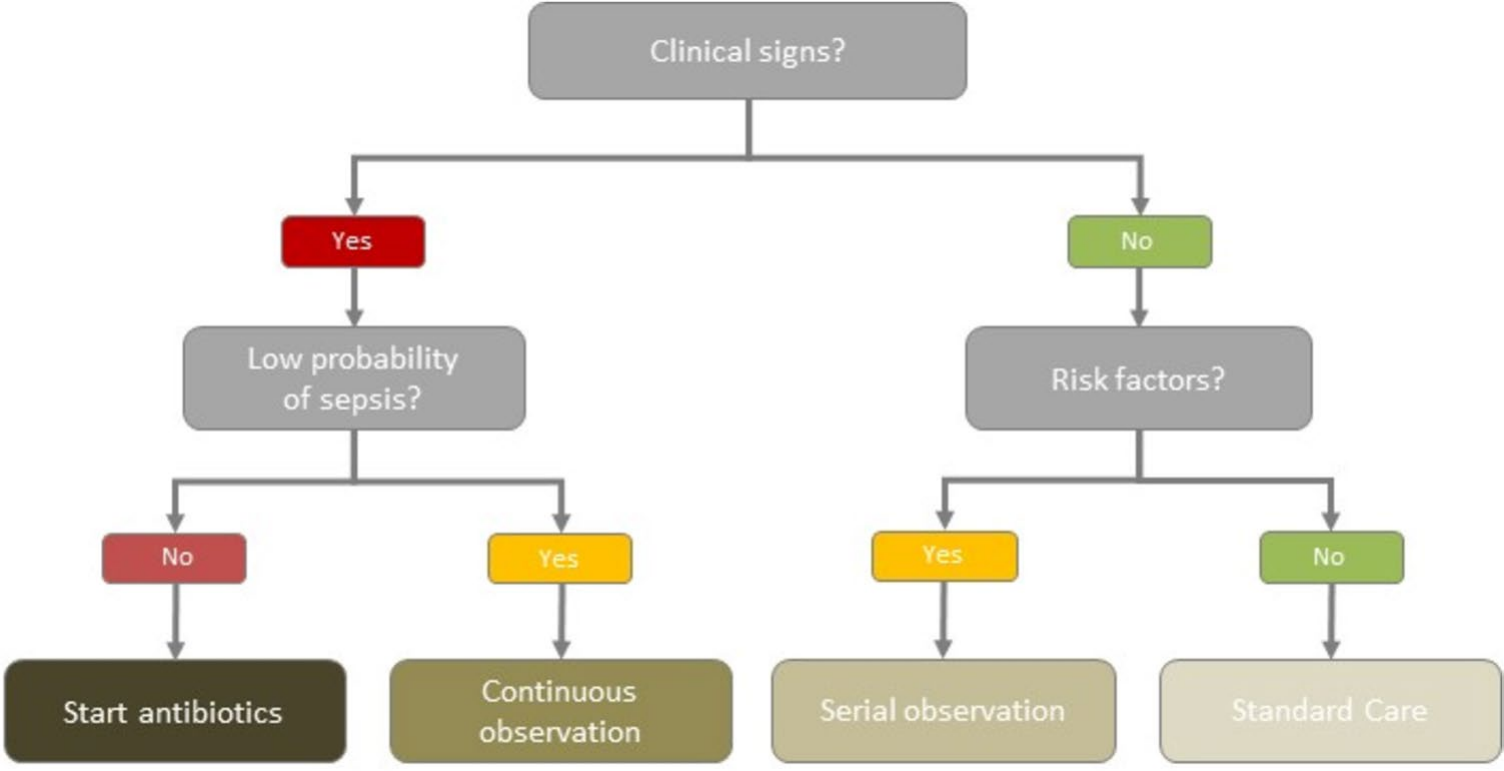
Algorithm to guide the management of newborns with clinical signs or risk factors potentially associated with early onset sepsis (EOS). Definitions and further information are provided in Table 1

**Fig. 2:**
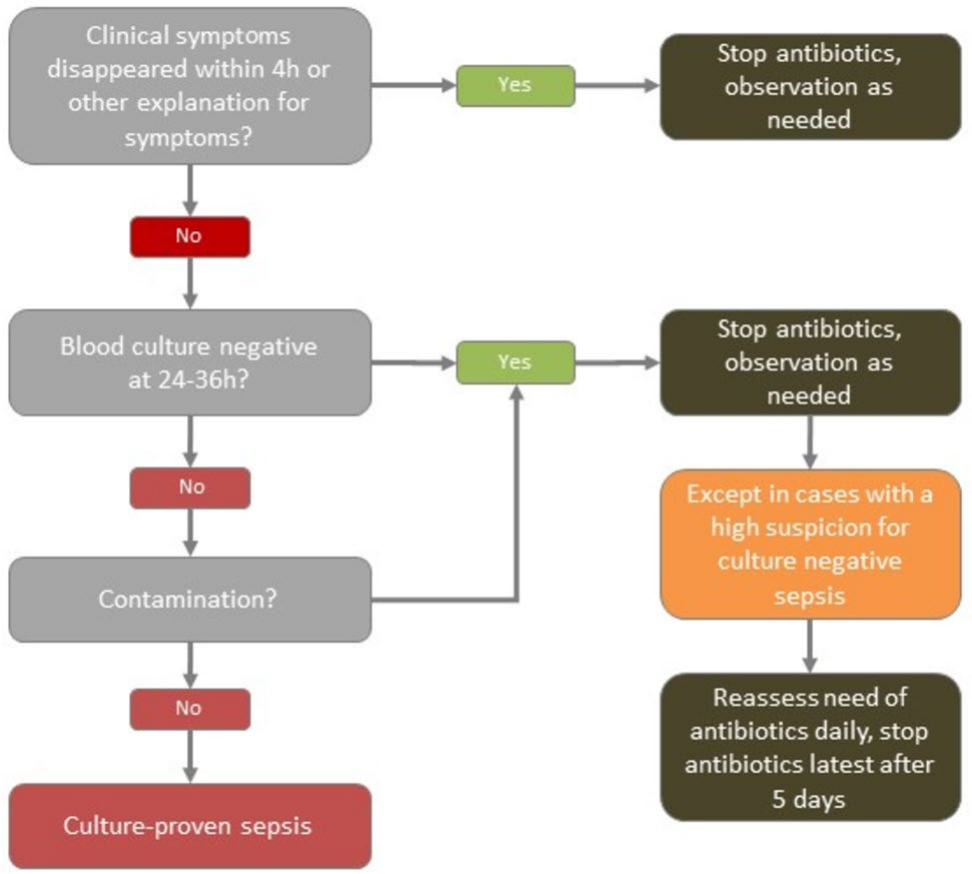
Algorithm to guide the duration of empirically started antibiotics. Definitions and further information are provided in Table 2

Figure 1 gives an overview of the algorithm regarding the observation of neonates at risk for EOS and the start of antibiotic therapy. Important definitions and further information are given in Table 1. Figure 2 and Table 2 entail an overview regarding management after the start of empirical antibiotics for suspected EOS.

**Table 2:** Definitions of contamination, culture-negative, and culture-positive neonatal early onset sepsis (EOS); information regarding the role of the dynamic aspect of clinical signs and biomarkers; and the need for lumbar puncture (compare Fig. 2)

Dynamic aspect of clinical signs	Progression of clinical signs points towards infection, whereas rapid resolution points towards no infection. Clinical deterioration within hours after delivery may be caused by a disease other than infection. If EOS is unlikely based on the course of clinical signs or there is an alternative explanation, consider to stop antibiotic therapy at any time point. To be able to stop antibiotic therapy early in situations with a low probability of EOS, serial assessments of the neonate are mandatory
Role of biomarkers	Serial negative PCT or CRP measurements may serve as an additional supporting argument for discontinuing antibiotic therapy in late preterm and term infants within 24 to 36 h. Increased biomarkers do not have a high positive predictive value for sepsis, and a diagnosis of culture-negative sepsis only due to increased biomarkers can lead to a substantial overuse of antibiotics
Lumbar puncture	We do not recommend performing a lumbar puncture on every neonate who is on antibiotic therapy. However, it should be considered and thoroughly discussed in all cases. Lumbar puncture is particularly recommended in cases with positive blood cultures, critical illness, or neurological symptoms such as seizures or altered consciousness. If the neonate’s condition does not permit the safe performance of a lumbar puncture prior to starting antibiotics, the procedure can be deferred. In such cases, cell count and a PCR test for meningitis in the LCR can be performed when the neonate is stable to inform and guide further management
Contamination	Pathogens in positive blood cultures have to be assessed regarding the possibility of contamination. Skin pathogens combined with a low probability of EOS point towards contamination, and antibiotics may be stopped in these situations. In situations with central catheters inserted during resuscitation, catheter-related infection and removal of the line have to be considered
Culture-positive sepsis	Length of antibiotic therapy in microbiologically documented infections depends on the pathogen, site of infection, and the clinical course of the patient. As soon as the pathogen is known, antibiotic treatment has to be targeted. Invasive infections with Gram-negative pathogens need a longer duration of treatment (14 to 21 days) compared to Gram-positive sepsis (7 to 14 days). In any case of culture-positive EOS, consultation with infectious disease specialists should be considered
Culture-negative sepsis	If blood cultures remain negative after 24 to 36 h, antibiotic therapy should be stopped. A culture-negative sepsis may be considered in neonates with multiple clinical signs, critical illness, and negative blood cultures. The probability of a culture-negative sepsis depends on the correct sampling of blood cultures: Blood cultures with a minimum of 1 ml blood sampled before the start of antibiotic therapy have an excellent sensitivity. In cases with a high suspicion for culture-negative sepsis, antibiotics should be discontinued latest after 5 days and not continued orally

## Data Availability

No datasets were generated or analysed during the current study.
